# Effects of cloprostenol sodium and carbetocin on synchronous parturition and colostrum composition in large white sows

**DOI:** 10.1186/s40813-025-00436-7

**Published:** 2025-04-16

**Authors:** Hongmei Gao, Miaolian Peng, Rongzhi Zhong, Zhenhua Xue, Zhenqiang Liu, Shiqiao Weng, Longchao Zhang, Dong Wang, Yan Liu, Jianhui Tian, Lixian Wang

**Affiliations:** 1 Institute of Animal Science, Chinese Academy of Agricultural Sciences, Beijing, 100193 People’s Republic of China; 2 Guangdong Wens Pig Breeding Technology Co. Ltd., Wens Foodstuff Group Co. Ltd., Yunfu, 527300 Guangdong People’s Republic of China; 3 The Municipal Animal Husbandry General Station of Beijing, Beijing, 100107 People’s Republic of China; 4 Ningbo Sansheng Biological Technology Co., Ltd., Ningbo, 315000 Zhejiang People’s Republic of China; 5 Institute of Animal Husbandry and Veterinary Medicine, Beijing Academy of Agriculture and Forestry Sciences, Beijing, 100097 People’s Republic of China; 6 Key Laboratory of Animal Genetics, Breeding and Reproduction of the Ministry of Agriculture and Rural Affairs; National Engineering Laboratory for Animal Breeding; College of Animal Science and Technology, China Agricultural University, Beijing, 100193 People’s Republic of China

**Keywords:** Cloprostenol sodium, Carbetocin, Large white sows, Farrowing induction, Colostrum

## Abstract

**Background:**

Synchronized parturition is essential for optimizing batch production and implementing effective "all-in/all-out" management strategies. However, the efficacy of exogenous hormones in regulating parturition and the potential adverse effects of parturition induction have remained inconsistent. This study investigates the impact of cloprostenol sodium and carbetocin on farrowing performance in sows, aiming to establish an optimal induction protocol.

**Results:**

Initially, based on a dataset comprising 3,657 gestation records of large white sows, we calculated the average gestation length for the herd to be 114 days, and the induction time was set at 113 days of gestation. Subsequently, parturition was induced in 546 primiparous sows and 339 multiparous sows, respectively. The experiment consisted of three groups: (1) CON group (control), (2) PG group (cloprostenol sodium), and (3) PGCAR group (combination of cloprostenol sodium and carbetocin). In primiparous sows, compared to the control group, the PG group exhibited farrowing rates of 23.5% on day 113 and 71.8% on day 114. Notably, the PGCAR group demonstrated a higher farrowing rate of 78.1% on day 114. Importantly, the working hours farrowing rate for the PGCAR group was 90.3%, with 64.6% of sows farrowing within five hours after carbetocin administration. Additionally, both the PG and PGCAR groups showed a significant reduction in farrowing duration, birth interval, and stillbirth rate compared to the control group. Similar trends were observed in multiparous sows. In the PG group, farrowing rates were 25.0% on day 113 and 66.7% on day 114. Notably, the PGCAR group demonstrated a farrowing rate of 76.2% on day 114. Specifically, within the PGCAR group, 87.7% of farrowing events occurred during working hours, and 68.0% of farrowing events occurred within five hours following carbetocin administration. Furthermore, compared to the control group, the PGCAR group of multiparous sows exhibited a significant reduction in both farrowing duration and birth intervals. Furthermore, our analysis revealed no statistically significant differences in colostrum composition and milk bioactive components across the CON, PG, and PGCAR groups.

**Conclusions:**

The combined administration of cloprostenol sodium and carbetocin effectively induces parturition in large white sows, accelerating the parturition process without adverse effects on either the sows or the piglets. However, 5–22% of the sows in this study exhibited spontaneous farrowing prior to hormone-induced parturition. Further investigation is necessary to elucidate the underlying mechanisms and address instances where sows initiate farrowing prematurely before induction.

## Background

With the expansion of the swine industry and the increasing disease challenges encountered by pig enterprises, more and more pig farmers are adopting batch management to supplant the traditional continuous production systems [1]. In the batch management, specific stages of the sow's reproductive cycle, such as mating, gestating, farrowing, and weaning, are synchronized at predetermined time point for facilitating efficient “all-in/all-out” management [2]. However, the batch farrowing may not always strictly adhere to the "all-in/all-out" strategy due to various factors, such as sow's environmental conditions, physiological state, and genetic variations, which can contribute to the variation in gestation length [3], typically ranging from 108 and 119 days [4]. Even if sows are synchronized in the same estrus and mated simultaneously, there may still be differences in their farrowing dates [5]. Furthermore, the farrowing process necessitates intensive supervision by workers to provide birthing assistance to sows [6]. Compounding this challenge, a significant proportion of sows exhibit nocturnal birthing patterns, which substantially increases the nighttime workload for staff. Unpredictable initiation of parturition, inadequate monitoring and care of birth is one of the reasons for the high rate of loss newborn piglets and stillbirth [7]. Moreover, sows with larger litter sizes experience extended parturition and compromised placental expulsion, which elevates the risk of postpartum metritis and delays uterine involution [8]. Therefore, the implementation of farrowing induction is necessary for batch farrowing in sows, as it enhances the predictability of farrowing timing and facilitates synchronized parturition during working hours [9]. Consequently, this enables a systematic arrangement of employees' work schedules, thereby improving their efficiency and ultimately achieving batch management [10].

The parturition process in most species is initiated by the release of prostaglandins (PG) and other inflammatory mediators. This release promotes the withdrawal of progesterone (P4) production while concurrently elevating estradiol (E2) levels. The resultant hormonal shift enhances uterine contractility, facilitates cervical dilation, and ultimately triggers parturition [11, 12]. The initiation of parturition in sows can often be achieved by regulating exogenous hormones that target the pregnant corpus luteum [13]. Currently, parturition induction in sows has been implemented in batch production, one of the most commonly utilized hormones is prostaglandin F2α (PGF2α) or its analogues, with a wealth of research available on induction protocols and strategies [14]. However, there still exist several challenges associated with achieving the expected synchronization effects [15], and the determination of the most optimal timing and dosage for administering PGF2α remains to be definitively established [16–18]. Additionally, combining PGF2α with oxytocin or carbetocin has been utilized to minimize the interval between induction and onset of parturition [19, 20]. Nevertheless, existing reports controversial, concerns have been raised that the improper administration of uterotonics may elevate the incidence of dystocia in sows, increase the proportion of stillborn piglets, and negatively impact the vitality of neonatal piglets [21–23], therefore, further research is necessary to investigate potential adverse effects of parturition induction.

The primary objective of this study is to optimize the utilization of exogenous hormones for synchronized parturition in sows, develop an efficient protocol for synchronous parturition, and investigate potential impacts on sow reproductive performance. Specifically, this research will examine factors including the concentration of working hours deliveries, duration of farrowing, stillbirth rates of piglets, and colostrum composition. Ultimately, these findings aim to provide valuable insights for further advancements in farrowing induction techniques.

## Materials and methods

### Animals and experimental design

The study was conducted on a commercial batch farm (Honghu, China), and the experimental protocols utilized in this study were approved by the Animal Care and Use Committee (No. IAS2022‐79) in accordance with the "Guide for the Care and Use of Laboratory Animals" edited by the Institute of Animal Sciences, Chinese Academy of Agricultural Sciences (Beijing, China). Initially, an analysis was conducted on the gestation records of 3657 large white sows in 2023 to determine the average gestation length for sows at this farm. Subsequently, an assessment was carried out on a total of 885 large white sows, including 546 primiparous sows and 339 multiparous sows with parities ranging from 2 to 7. The first artificial insemination (AI) was designated as day zero ('0') of gestation. Gestation length refers to the period from insemination until farrowing, encompassing the entire duration of pregnancy. All sows are vaccinated in strict compliance with the farm's established management protocols, and the ambient temperature within the enclosures is maintained between 22 and 25 °C. Sows are provided with 2.5 kg of commercial feed daily until day 111 of gestation, after which the feed allowance is increased to 3.5 kg per day until parturition. Gestating sows are housed individually in stalls, and approximately seven days prior to their expected farrowing date, they are transferred to individual farrowing pens. Each facility is equipped with an automatic feeder and sows have unrestricted access to water.

Sows were classified into primiparous and multiparous categories based on their parturition experience. Each category of sows was randomly assigned to one of three treatments: (1) CON group—sows farrowed spontaneously without exogenous hormonal interventions; (2) PG group—sows received a perivulval injection of cloprostenol sodium (0.1 mg, Ningbo Sansheng Biotechnology Co., LTD., China) at 07:00 am one day prior to the expected farrowing date. Sows that farrowed prior to the administration of cloprostenol sodium were documented accordingly; (3) PGCAR group—sows were initially treated with cloprostenol sodium the same as PG group, followed by intramuscular injections of carbetocin (35 µg, Ningbo Sansheng Biotechnology Co., LTD., China) after 24 h. Sows that farrowed before the administration of carbetocin were accurately recorded. Continuous supervision was ensured for all sows throughout the entire farrowing process, all piglets were individually identified through the application of an ear tag immediately after birth.

### Data collection

The recorded parameters of the sows included gestation length, total number of piglets born per litter (TB), number of piglets born alive per litter (BA), number of stillborn per litter (ST), and mummified foetuses per litter (MU). Farrowing duration (FD), defined as the time interval between the expulsion of the first and last piglets, and birth interval (BI) were also measured. The working hours were defined as between 07:00 and 16:59. Birth assistance is provided when necessary to examine the birth canal for potential obstructions, particularly if the interval between the delivery of two piglets exceeds 30 min.

### Colostrum collection and composition assay

Colostrum samples were collected from 10 sows, comprising 5 primiparous sows and 5 multiparous sows with parities ranging from 2 to 5 in each treatment group, with a volume of 30 ml obtained within 6 h after the first piglet was farrowed. The milk samples were divided into two groups: 20 ml of the samples were stored at 4 °C for composition analysis, while the remaining samples were centrifugated at 2000 × g for 15 min to extract whey. The extracted whey was subsequently stored at − 20 °C for detection of active substances.

Milk composition analysis, including fat, protein, lactose, total solids (TS), and non-fat solids (SNF), was conducted using the Fourier Transform Infrared Spectroscopy (FTIR) MilkoScan™ FT3 automatic milk analyzer (Foss Company, Denmark).

The concentrations of immunoglobulin A (IgA), immunoglobulin G (IgG), immunoglobulin M (IgM), insulin (INS), growth hormone (GH), interleukin-1β (IL1β), interleukin-6 (IL6), tumor necrosis factor α (TNF-α) and cortisol in whey were determined using ELISA kit (Gene Lab Biotechnology Co., Ltd., Beijing China) according to the instructions.

### Statistical analyses

The statistical analyses were conducted using the statistical analysis system software (SAS®, version 9.4, Institute Inc., Cary, NC, USA). Descriptive statistics for sow gestation length including mean values and ranges were generated using the MEANS procedure of SAS. For sow variables and colostrum composition parameters, a general linear model (GLM) procedure was employed for analysis. The statistical models incorporated the treatment groups (CON, PG, and PGCAR) as fixed effects and the residual term as a random effect. The results were expressed as least squares means (LSmeans) ± standard error of the mean or percentages. The incidence of birth assistance rate, characterized by binomial traits, was analyzed utilizing the GENMOD procedure of SAS. Descriptive statistics significance was considered at *P* ≤ 0.05.

## Results

### Determination of the optimal timing for inducing parturition in sows

**Fig. 1:**
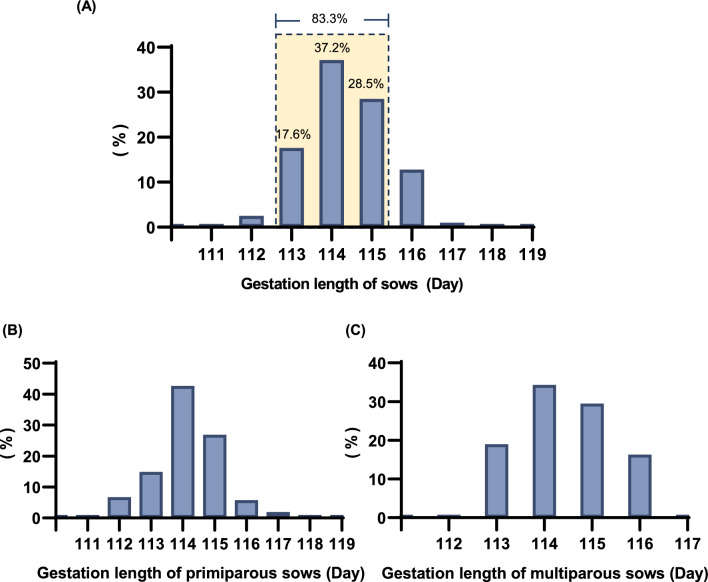
The distribution of gestation length among large white sows. **A** The distribution of gestation length of all sows. **B** The distribution of gestation length of primiparous sows. **C** The distribution of gestation length of multiparous sows

Based on the gestation records of 3,657 non-induced farrowing large white sows from the farm in 2023, we have calculated that the average gestation length for the herd was 114.3 days, with sows exhibiting a range of gestational lengths from 111 to 119 days. Subsequently, an analysis of the proportion of gestation length among sows revealed that approximately 83.3% delivered between days 113 and 115, with a subset of 37.2% farrowing specifically on day 114 (Fig. 1A). Considering that multiparous sows possess prior parturition experience and exhibit greater physiologically and behaviorally maturity compared to primiparous sows, this may result in differences in gestation length between the two groups. We categorized the sows into primiparous and multiparous groups to calculate their respective gestation lengths. The results indicated that the average gestation length for primiparous sows was 114.2 days, while that for multiparous sows was 114.5 days, which is marginally longer. Moreover, the gestation periods for multiparous groups were relatively concentrated within the range of 112 to 117 days (Fig. 1B, 1C and Table 1). Therefore, it can be inferred that the expected farrowing date for both primiparous and multiparous sows within this particular herd is approximately day 114. Consequently, in this experiment the induction of parturition will be conducted one day prior to the expected farrowing date (i.e., 113 days).

**Table 1:** Descriptive statistics of sow gestation length

	All sows	Primiparous sows	Multiparous sows
Number	3657	1243	2414
Gestation length, day	114.3 ± 13.0	114.2 ± 13.9	114.5 ± 29.4
Range, day	111–119	111–119	112–117

### The impact of exogenous hormones on synchronized parturition in primiparous and multiparous sows

**Fig. 2:**
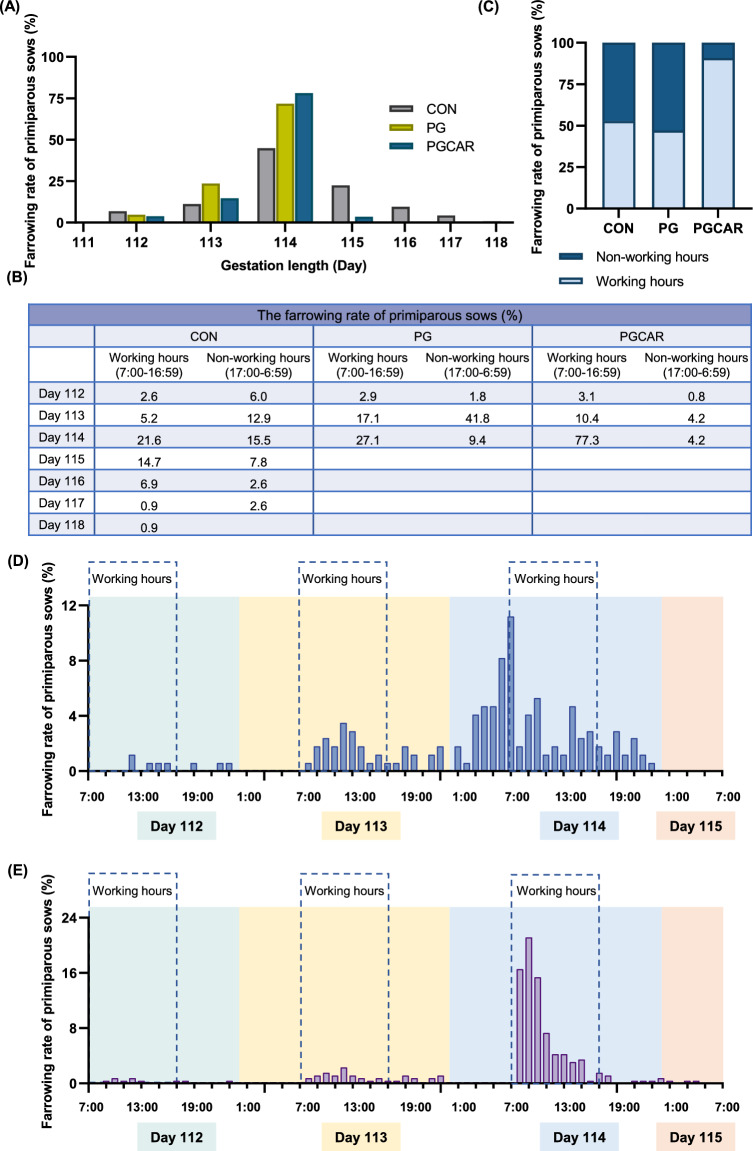
The timing of parturition in primiparous sows. **A** The gestation length of primiparous sows in the CON, PG and PGCAR groups. **B** The farrowing rates of sows during working hours (07:00 to 16:59) and non-working hours (17:00 to 6:59) for the CON, PG, and PGCAR groups on a daily basis. **C** The total farrowing rates of sows during working hours (07:00 to 16:59) across the three group. **D** The hourly farrowing frequencies of sows in the PG group during days 112–115 of gestation. **E** The hourly farrowing frequencies of sows in the PGCAR group during days 112–115 of gestation

The impact of cloprostenol sodium and carbetocin on the synchronized parturition was assessed by measuring the time onset of farrowing in each sow. Considering the divergent maternal behaviors of primiparous sows and multiparous sows, we first analyzed the gestation length of primiparous sows in each experimental group. As depicted in Fig. 2A, the sows in the control group farrowed spontaneously, exhibiting varying farrowing rates within the gestation period of 112–118 days, and the highest farrowing rate (44.8%) was observed on day 114. The primiparous sows in both the PG and PGCAR groups achieved parturition within a gestation length of 115 days. The sows in the PG group primarily farrowed on the 113th and 114th day, with respective rates of 23.5% and 71.8%. The PGCAR group, in contrast, exhibited a higher farrowing rate of 78.1% on the 114th day. The farrowing rate of sows during working hours (7:00 a.m. to 16:59 p.m.) and non-working hours (17:00 p.m. to 6:59 a.m.) for each group was calculated on a daily basis depicted in Fig. 2B. The total farrowing rates of sows during working hours across the three group were presented in Fig. 2C, demonstrating percentages of 52.8%, 47.1%, and 90.3% respectively. Additionally, Fig. 2D illustrates the hourly farrowing frequencies of primiparous sows in the PG group during days 112–115 of gestation, indicating a dispersed distribution of farrowing events among sows throughout this period. Similarly, Fig. 2E displays the hourly farrowing frequencies of sows in the PGCAR group on days 112–115 of gestation. Importantly, there was a significant surge in farrowing rate within four hours following carbetocin injection, specifically with the peak reaching at 64.6% between 7:00 and 11:59.

**Fig. 3:**
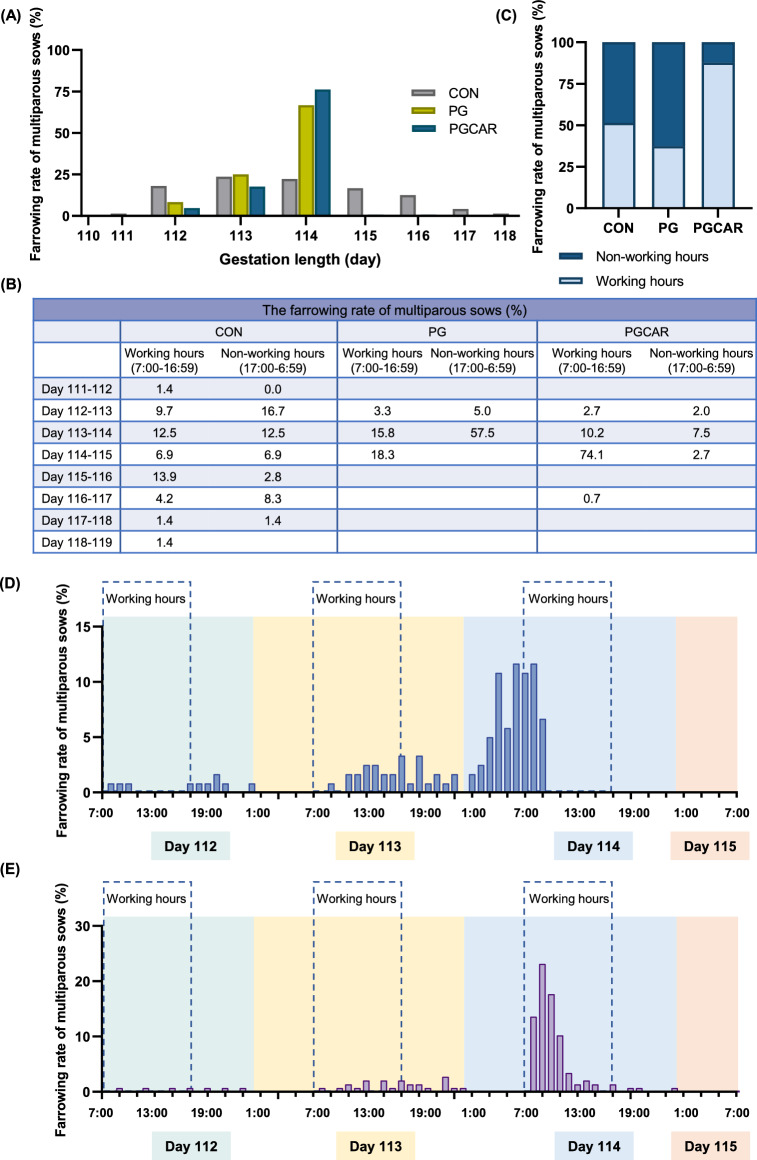
The timing of parturition in multiparous sows. **A** The gestation length of multiparous sows in the CON, PG and PGCAR groups. **B** The farrowing rates of sows during working hours (07:00 to 16:59) and non-working hours (17:00 to 6:59) for the CON, PG, and PGCAR groups on a daily basis. **C** The total farrowing rates of sows during working hours (07:00 to 16:59) across the three group. **D** The hourly farrowing frequencies of sows in the PG group during days 112–115 of gestation. **E** The hourly farrowing frequencies of sows in the PGCAR group during days 112–115 of gestation

We subsequently conducted an analysis on multiparous sows. Within the control group, the gestation length distribution ranged from 111 to 118 days, with higher farrowing rates observed on days 113 and 114 (23.6% and 22.1%, respectively). The majority sows in the PG group farrowing on days 113 and 114, accounting for 25.0% and 66.7%, respectively. While sows in the PGCAR group reached a remarkable high farrowing rate of 76.2% on day 114 (Fig. 3A). The Fig. 3B and C illustrate the farrowing rates for sows in each treatment group, both during working hours and non-working hours. It is worth noting that the PGCAR group not only demonstrated synchronized farrowing on the 114th day, but also achieved a farrowing rate of up to 87.7% during working hours. Additionally, Fig. 3D and E depict the hourly farrowing frequencies of multiparous sows both in the PG and PGCAR groups during days 112–115 of gestation, respectively. The findings obtained for multiparous sows were consistent with those observed in primiparous sows, within five hours after carbetocin injection, the farrowing rate of sows in the PGCAR group reached a remarkable level of 68.0%.

### Effects of exogenous hormones on the performance of primiparous and multiparous sows

When analyzing the PG and PGCAR groups in both primiparous and multiparous sows, we excluded sows that failed to complete the hormone induction protocol in each group. Specifically, among the primiparous sows, eight sows in the PG group did not receive cloprostenol sodium injection, ten sows in the PGCAR group did not receive cloprostenol sodium injection, and thirty-eight sows did not receive carbetocin injection. The effects of cloprostenol sodium and carbetocin on the performance of primiparous sows are summarized in Table 2. It demonstrates a significant reduction in farrowing duration and birth interval for both the PG and PGCAR groups compared to the control group. Additionally, the PG and PGCAR groups exhibited a significant decrease in stillbirth rates among sows relative to the control group. Furthermore, the PG group displayed a significantly higher total number of piglets born (TB) compared to the PGCAR group, while alive born (AB) from the PG group surpassed those from both the CON and PGCAR groups. No significant differences were observed among the three groups regarding the rates of mummified piglets and the incidence of birth assistance.

**Table 2:** The impact of cloprostenol sodium and carbetocin on the performance of primiparous sows

Variables	Without induced parturition	Induced parturition	
	CON	PG	PGCAR
Total number of sows	116	170	260
Number of sows excluded	0	8	10 + 38
Number of experimental sows	116	162	212
Gestation length, day	114.3 ± 0.06^a^	113.7 ± 0.05^b^	114.0 ± 0.04^c^
Farrowing duration, min	174.9 ± 5.65^a^	158.9 ± 4.78^b^	152.4 ± 4.18^b^
Birth interval, min	13.2 ± 0.44^a^	11.3 ± 0.37^b^	11.5 ± 0.33^b^
Total born	13.8 ± 0.27^ac^	14.4 ± 0.23^a^	13.7 ± 0.20^bc^
Alive born	11.1 ± 0.27^a^	12.71 ± 0.23^b^	11.7 ± 0.20^a^
Stillborn piglets, %	10.8 ± 0.77^a^	6.5 ± 0.66^b^	6.7 ± 0.57^b^
Mummified fetues, %	7.1 ± 0.84	5.4 ± 0.71	7.5 ± 0.62
Birth assistance, %	12.9	11.7	13.7

^a,b,c^Different lowercase letter superscripts in the same column indicate significant difference (*P* < 0.05)

Similarly, an analysis of multiparous sows was presented in Table 3. Among the multiparous sows, ten sows in the PG group did not receive cloprostenol sodium injection, seven sows in the PGCAR group did not receive cloprostenol sodium injection, and twenty-six sows did not receive carbetocin injection. It is evident that the PGCAR group significantly reduced both farrowing duration and birth intervals compared to the control group. However, no statistically significant differences were observed among the three groups with respect to the total number of piglets born, the number of live births, stillbirth rates, mummified piglet rates, or the rate of birth assistance.

**Table 3:** The impact of cloprostenol sodium and carbetocin on the performance of multiparous sows

Variables	Without induced parturition	Induced parturition	
	CON	PG	PGCAR
Total number of sows	72	120	147
Number of sows excluded	0	10	7 + 26
Number of experimental sows	72	110	114
Parity	3.6 ± 0.19	3.4 ± 0.15	3.2 ± 0.15
Gestation length, day	114.0 ± 0.10	114.2 ± 0.08	114.0 ± 0.08
Farrowing duration, min	192.6 ± 6.61^a^	177.9 ± 5.35^a^	156.3 ± 5.25^b^
Birth interval, min	13.0 ± 0.51^a^	11.9 ± 0.42 ^ab^	10.9 ± 0.41^b^
Total born	15.4 ± 0.37	15.4 ± 0.30	14.6 ± 0.29
Alive born	13.3 ± 0.35	13.1 ± 0.29	12.5 ± 0.28
Stillborn piglets, %	7.8 ± 0.89	7.2 ± 0.72	6.1 ± 0.70
Mummified fetues, %	5.4 ± 1.03	7.9 ± 0.83	8.0 ± 0.82
Birth assistance, %	8.3	9.1	8.7

^a,b,c^Different lowercase letter superscripts in the same column indicate significant difference (*P* < 0.05)

### Effects of exogenous hormones on the composition of colostrum

**Fig. 4:**
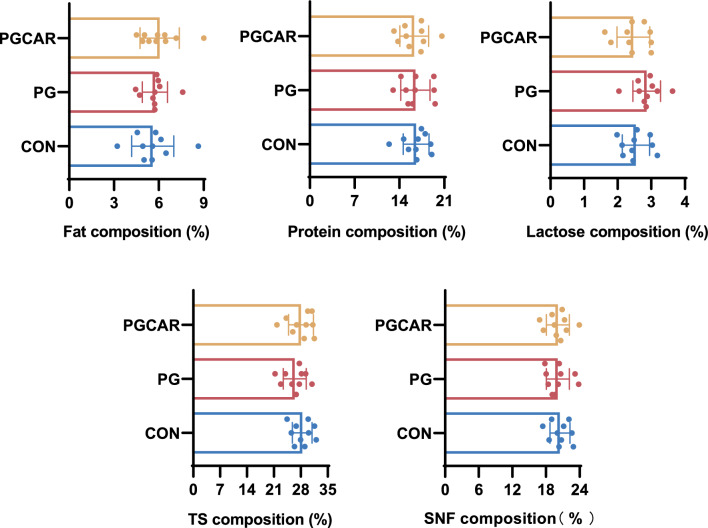
The effects of cloprostenol sodium and carbetocin on protein, fat, lactose, total solids (TS), and non-fat solids (SNF) content in colostrum samples from sows in the CON, PG, and PGCAR groups

A total of five primiparous sows and five multiparous sows were selected from each treatment group for the evaluation of milk composition. The colostrum composition exhibited overall similarity between primiparous and multiparous sows, leading to the combination of data from both categories for analysis. No significant differences were observed in the protein, fat, lactose, total solids (TS), and non-fat solids (SNF) content of colostrum samples across the three groups (Fig. 4).

**Fig. 5:**
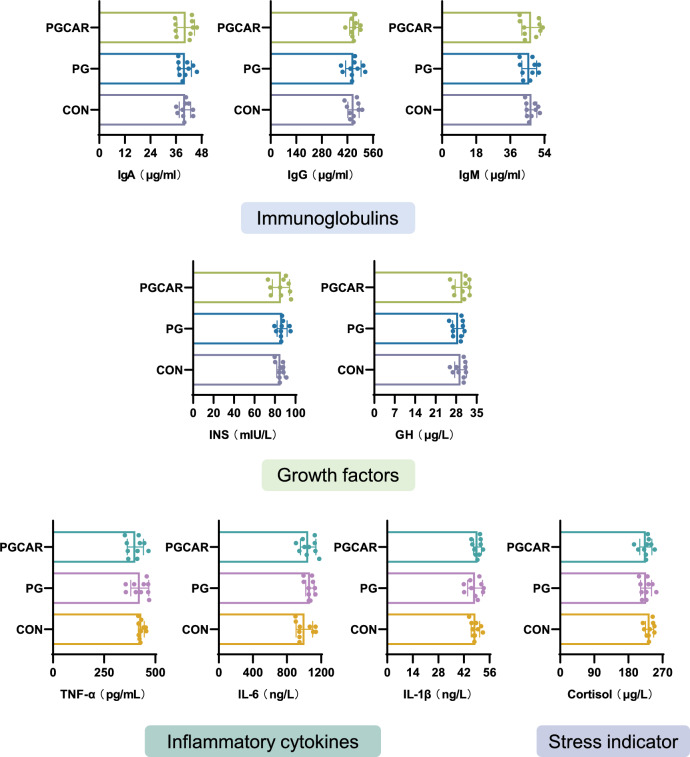
The impact of cloprostenol sodium and carbetocin on the concentrations of bioactive compounds, including immunoglobulins (IgA, IgM, and IgG), growth factors (INS and GH), inflammatory cytokines (IL-1β, IL-6, and TNF-α), and cortisol, in colostrum samples from sows in the CON, PG, and PGCAR groups

Additionally, no significant variations were noted in the concentrations of bioactive substances, including immunoglobulins (IgA, IgM, and IgG) and growth factors (INS, GH), which are essential for piglet growth and development. Furthermore, the levels of interleukin-1β (IL1β), interleukin-6 (IL6), tumor necrosis factor α (TNF-α), and cortisol were quantified, and the results indicated no significant differences in inflammatory cytokines and cortisol levels among the three groups (Fig. 5).

## Discussion

The parturition induction is not only crucial in the batch production of sows but also ensures efficient, safe, and scientifically managed delivery of piglets, thereby enhancing the reproductive efficiency of the sows [24]. Nonetheless, numerous challenges persist in achieving optimal efficacy during synchronized parturition. It is widely recognized that inappropriate drug administration can exert adverse effects on both sows and piglets. Therefore, this study investigates the synchronization effects of cloprostenol sodium and carbetocin on farrowing performance of sows, aiming to standardize synchronized parturition and provide a theoretical foundation for further batch production in pig farms.

The precise definition of gestational day "0" and the accurate estimation of the expected farrowing date are essential for the accurate evaluation of parturition induction and synchronization protocols [25]. The gestation period of pigs varies from 108 to 119 days due to variations in pig breeds and farm management practices [26]. However, the availability of comprehensive and precise information regarding the definition of gestational day "0", estimation of expected farrowing dates, and determination of synchronized induction timing remains limited in most research. Instead, a fixed time is typically chosen for inducing parturition, which can have varying effects on pig herds with different gestation periods. Specifically, sows with shorter gestation length may initiate parturition before the prostaglandin takes effect, while inducing parturition too early for sows with longer gestation length may be detrimental [27, 28]. Furthermore, when inducing parturition, consideration should be given to the lung developmental stage of pig fetuses [29]. According to reports, alveoli development in pig fetuses is not fully complete until 113 days [30], and premature induction of parturition could lead to severe consequences such as impaired lung development and postnatal mortality [31]. In this experiment, the initial artificial insemination (AI) was designated as gestational day 0. Based on historical farm records, the average gestation length of the pig herd was estimated to be approximately 114 days. In accordance with the principle of not inducing parturition earlier than 113 days' gestation [32], the induction of parturition for the pig herd was scheduled for day 113.

Given that the maternal behavior of sows can be influenced by previous experience and considering the reported association between parity and maternal performance [33], we categorized sows into primiparous and multiparous based on their parities. Compared to primiparous sows experiencing parturition for the first time, multiparous sows, with prior experience in motherhood, exhibit greater physiological, behavioral, and immunological maturity. This difference may influence both farrowing performance and colostrum production between primiparous and multiparous sows [34]. This classification enables us to investigate the distinct responses of different parities to exogenous hormones. When comparing the CON group of primiparous and multiparous sows, it is evident that the farrowing rate before the expected date (114 days) was 18.1% and 43.1%, respectively. Furthermore, it can be observed that primiparous sows exhibit a delayed initiation of farrowing compared to multiparous sows, highlighting the importance of considering parity when determining the optimal timing for inducing farrowing. However, consistent findings were noted among both primiparous and multiparous sows in subsequent PG and PCCAR groups, implying the feasibility of an induced protocol at 113 days. Nevertheless, during our experiment, some sows in both the PG group and the PGCAR group farrowed before completing the hormone treatment. We included this data in our results as excluded sows, which comprised 5 to 22% of the total experimental groups. Therefore, further advancements are necessary to address instances when sow farrowing occurs prior to day 113.

In this experiment, administering cloprostenol sodium to induce parturition significantly increased the degree of parturition synchronization as expected. Compared to the control group, both primiparous and multiparous sows in the PG group concentrated parturition at 113 days and 114 days of gestation. However, to address the issue of scheduling farrowing for sows during working hours, sows in the PG group had lower farrowing rates than those in the PGCAR group. The termination of pregnancy by prostaglandin occurs through the dissolution of the corpus luteum and reduction in progesterone levels [35]. However, it is important to note that progesterone withdrawal constitutes the pivotal event in hormonal regulation, which is triggered by the integration of intricate endocrine or paracrine signals within the physiological context [36]. This may elucidate why prostaglandins alone fail to achieve the desired induction effect. In this study, we implemented a drug regimen combining cloprostenol sodium and carbetocin to induce synchronized parturition in sows. Carbetocin, an extended-acting analogue of oxytocin that shares the same receptor, stimulates uterine smooth muscle contraction by triggering the release of abundant free calcium ions from the endoplasmic reticulum [37]. Compared to oxytocin, carbetocin exhibits higher receptor affinity, longer half-life, faster onset of action, and more sustained effects, making it a potential safe and effective alternative in the future [38]. In this study, the PGCAR group effectively achieved synchronization of parturition at 114 days in both primiparous and multiparous sows, with farrowing rates reaching 78.1% and 76.2%, respectively. Additionally, the analysis of working hours revealed that the combination regimen significantly enhanced farrowing rates to 90.3% in primiparous sows and 87.7% in multiparous sows. Furthermore, 64–68% of sows in the PGCAR group exhibited onset of farrowing within five hours following carbetocin injection, thereby enhancing the efficiency of farrowing supervision.

The combination of cloprostenol sodium and carbetocin offers a promising alternative for inducing farrowing in sows. Given the detrimental effect of oxytocin's uterine contractions on piglet mortality, although carbetocin demonstrates a comparatively lower intensity and shorter duration of contractions in comparison to oxytocin [39], further investigation is still warranted to ascertain whether carbetocin presents similar adverse effects, particularly when extensively employed in batch production. We conducted an analysis on the impact of cloprostenol sodium and carbetocin on the reproductive performance of sows, with a specific focus on their effects on farrowing duration, birth interval, and stillbirth rate. The duration of parturition is a critical factor that determines the survival of piglets, prolonged farrowing duration in sows increases the risk of piglet mortality due to asphyxia [40, 41]. Stillbirth poses significant economic and welfare challenges in pig farming [42]. Previous studies have established a direct correlation between farrowing duration and the incidence of stillbirth [43]. In the primiparous group of this study, both the PG and PGCAR group exhibited significantly shorter farrowing duration, birth interval, and stillbirth rates compared to the control group. Among multiparous sows’ groups, only the PGCAR group showed significantly shorter farrowing duration and birth interval compared to the control group; however, there was no significant difference observed in stillbirth rates between these two groups although a downward trend in stillbirth rates within the PGCAR group was noted as well. These experiments demonstrate that cloprostenol sodium and carbetocin do not negatively impact sow reproductive performance; instead, they concentrate parturition rates while effectively shortening farrowing durations thereby reducing incidences of stillbirth.

Colostrum serves as a valuable source of vital nutrients and bioactive compounds, which play a pivotal role in providing energy, facilitating early gastrointestinal maturation, and particularly offering passive immune protection to neonatal piglets [44–46]. The synthesis of colostrum is primarily regulated by the coordination of reproductive and metabolic hormones [47]. Therefore, this study aimed to investigate the impact of exogenous hormones on the components of colostrum. Our analysis revealed no statistically significant differences in colostrum composition, bioactive substances (including immunoglobulins IgA, IgM, IgG and growth factors INS, GH), inflammatory cytokine markers (IL-1β, IL-6, TNF-α), and stress marker cortisol levels among the CON, PG and PGCAR groups. These findings confirm that cloprostenol sodium and carbetocin have minimal impact on sow's colostrum composition. However, previous research has indicated that the administration of PGF2α to induce parturition does not influence colostrum yield [48, 49]. Conversely, the use of carbetocin may adversely effect on colostrum production in sows. This negative impact could be attributed to carbetocin's potential to competitively bind to oxytocin receptors, thereby interfering with the normal binding of oxytocin to its receptors and consequently disrupting colostrum production [50]. Even when carbetocin is administered after the birth of the first piglet [51] or during the mid-stage of parturition, this adverse effect may still persist [52]. The measurement of colostrum yield is not solely determined by the entire litter piglet intake, but primarily relies on the sow's production capacity [53]. Various factors such as nutrition, hormones, and environment can all impact colostrum production in sows [54]. Further research is imperative to comprehend the extent to which farrowing induction affects the variability of colostrum yield and elucidate the regulatory mechanism underlying colostrum production in sows. Based on these findings, it is advisable to implement close supervision and care for neonatal piglets following carbetocin treatment. Where feasible, efforts should be made to enhance colostrum intake through the provision of additional colostrum or energy supplements [55].

## Conclusions

The induction of farrowing is pivotal in enhancing the production efficiency and management standards of pig farms. Nevertheless, it is essential to carefully consider the potential adverse effects impacts on sows and piglets when implementing synchronized farrowing. This study illustrates that a combination protocol, consisting of perivulval administration of cloprostenol sodium at 113 days of gestation followed by a subcutaneous injection of carbetocin 24 h later, can effectively induce farrowing in large white sows with an average gestation length of 114 days. This method not only streamlines work schedules and concentrates farrowing but also accelerates the parturition process without adverse effects on sows and piglets. Additionally, synchronized farrowing should be closely coordinated with farrowing supervision to ensure timely and appropriate care for both sows and neonatal piglets, thereby facilitating the effective implementation of batch synchronized farrowing. However, in this study, 5–22% of the sows initiated farrowing prior to hormone-induced parturition. Further research is warranted to investigate the causes of premature farrowing before induction and develop strategies to mitigate such occurrences.

## Data Availability

All data generated or analyzed during this study are included in this published article.

## Abbreviations

PG: Prostaglandins
P4: Progesterone
OT: Oxytocin
PGF2α: Prostaglandin F2α
TB: Total number of piglets born per litter
BA: Number of piglets born alive per litter
ST: Number of stillborn per litter
MU: Mummified foetuses per litter
FD: Farrowing duration
BI: Birth interval
TS: Total solids
SNF: Non-fat solids
IgA: Immunoglobulin A
IgG: Immunoglobulin G
IgM: Immunoglobulin M
INS: Insulin
GH: Growth hormone
IL-1β: Interleukin-1β
IL-6: Interleukin-6
TNF-α: Tumor necrosis factor α
GLM: General linear model
ANOVA: Analysis of variance
